# Enhanced optical transmission and Fano resonance through a nanostructured metal thin film

**DOI:** 10.1038/srep10393

**Published:** 2015-05-18

**Authors:** Bo Xiao, Sangram K. Pradhan, Kevin C. Santiago, Gugu N. Rutherford, Aswini K. Pradhan

**Affiliations:** 1 Department of Engineering and Center for Materials Research, Norfolk State University, Norfolk, VA 23504, USA

## Abstract

Artificial and engineered nanostructures expand the degrees of freedom with which one can manipulate the intricate interplay of light and matter. Certain nanostructural arrangements in the excited state enable the efficient electromagnetic coupling of propagating light with localized fields. Here, we demonstrate that light transmitted through a nanostructured metal thin film without any apertures can be significantly enhanced. Distinct asymmetric Fano resonances are observed in the zero-order transmission spectra using an incoherent light source. The transmission efficiency surpasses that of a metal thin film with the same area and thickness at the resonance maxima. The transmission minima and the sharp resonance maxima bear a strong resemblance to the extraordinary optical transmission observed in sub-wavelength nanohole array structures The resonance wavelength closely matches the nanostructural periodicity. The sensitivity of the resonances to the surrounding medium and the transmission efficiency demonstrate the potential for use in energy harvesting, imaging, optical processing and sensing applications.

When a nanoparticle is illuminated by a beam of light, observations made in the forward direction correspond to a superposition of the incident beam and forward-scattered electromagnetic fields. The spatial distribution and quantity of light both scattered and absorbed depend on the particle composition, size and shape. Advances in nanoscience and nanotechnology are producing an increasing number of methods for the manipulation of these parameters at the nanoscale. Recently, light transmission through a subwavelength aperture or an array of such apertures, such as nanoholes^1^,^2^ and nanoslits^3^,^4^, has been extensively studied, and these studies have revealed the intricate manipulation of interactions between light and nanostructures. The enhanced resonant transmission is not a unique phenomenon in the perforated metal thin films. Corrugated metal films or flat metal films with periodically arranged nanostructures can excite plasmon resonances at both sides of the films, which result similar transmission effects^5^,^6^,^7^. Central to this manipulation is the role of surface plasmon resonance, which is the collective oscillation of electrons bound to a metallic surface. Although studies of the role played by plasmon resonance in field enhancement are still ongoing^8^,^9^,^10^,^11^, the slow progress toward a full understanding of these phenomena has not hindered their growing use in a variety of imaging^12^,^13^,^14^ and sensing^15^,^16^ applications. Nevertheless, the engineering of metallic nanostructures seems to unambiguously provide a suitable paradigm for the manipulation of electromagnetic waves across the entire solar spectrum and beyond. The identification and construction of nanostructures that may effectively and efficiently confine or enhance electromagnetic fields to produce desired radiation distributions pose an unrelenting challenge. Here, we demonstrate that light transmission can be significantly enhanced at a specific wavelength using a properly nanostructured metal thin film without any apertures. The high coupling efficiency produces distinct light–matter interactions, which manifest as sharp Fano resonances in the transmission spectra.

Current nanofabrication technology offers numerous methods of arranging nanostructures with certain symmetries. However, the disparity among individual entities in a nanostructured system is seemingly neglected and often presumed to be negligible. Unfortunately, it is extremely difficult to create perfectly identical structures at the nanometer scale. Furthermore, nanofabrication processes such as lift-off techniques and dry etching unavoidably introduce sharp edges, corners and rough surfaces that cause unwanted losses. However, these transition losses can be minimized by shaping plasmonic structures with slow or adiabatic transitions rather than abrupt ones^17^,^18^,^19^,^20^. One key element of our approach is the alleviation of structural discrepancies by smoothing sharp edges, corners and rough surfaces. For this purpose, a simple method is developed for the formation of a nanostructured metal thin film based on the following steps: fabricating the nanostructures, reshaping the surface profile, and coating the surface with a thin metal layer. Hence, a complete structure generally consists of three layers: a nanowire array, a dielectric layer and a metal thin film (Fig. 1A). Varying the periodicity of the nanowire array modifies the resonance frequency/wavelength of this nanostructured system. Nanowire arrays are fabricated on a glass substrate by electron beam lithography. A thin dielectric layer of polymethyl methacrylate (PMMA) is spin coated onto the nanowire-array-patterned substrate and then thermally treated at an elevated temperature (~180 °C) to round the corners and edges and to smooth the surfaces. In the final step, a thin metal layer is deposited by electron beam evaporation. Gold, silver or a combination of gold and silver can be used for the nanowire array or the top metal thin-film layer in these nanostructures. We observed similar phenomena using both metals. Typically, the thickness of the top metal layer and the height of the nanowires are both approximately 40 nm. The widths of the nanowires are controlled to fall within the subwavelength range, from 60 to 90 nm. The coating and baking of the PMMA produces a smooth, curved layer covering the nanowires, which determines the final topography of the top metal thin film. The cross-sectional profile of such a structure is visible in the scanning electron microscope (SEM) images presented in Fig. 1 B,C.

The zero-order optical transmission spectra of the structure were measured using an ultraviolet–near-infrared spectrophotometer and an incoherent white light source. The optical transmission efficiencies relative to an equivalent area of a metal thin film of the same thickness were obtained for transverse magnetic light (TM-polarized, with the electric field perpendicular to the direction of the nanowire grating) at normal incidence. Unusually high transmission peaks with a distinct asymmetric Fano resonance line shape were observed for these nanostructures; nanostructures with various periods from 500 to 700 nm were investigated, and in each case, the resonance wavelength was 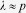 (where *p* is the nanowire period) (Fig. 2A). The wave vectors of the surface plasmons in our nanowire array structures should also satisfy the momentum-matching condition 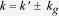, where *k* is the surface plasmon wave vector, *k′* is the component of the wave vector of the incident light that is parallel to the surface and perpendicular to the nanowire grating, and *k*_*g*_* = 2π/p* (where *p* is the nanowire period) is the wave vector of the metal grating. The transmission efficiencies observed at the resonance maxima surpassed those for a metal thin film with the same fraction and thickness. Additionally, no such phenomenon was observed in the transmission spectra under illumination with transverse electric light (TE-polarized, with the electric field parallel to the direction of the nanowire grating). The transmission spectra exhibited extraordinary optical transmission (EOT) surprisingly similar to that observed for subwavelength nanohole arrays^1^, which exhibit asymmetric Fano resonances with a strong dependence on the array periodicity and light polarization. In addition, strong coherent responses were revealed by the sharp resonance peaks, the full width half maxima (FWHM) of which were below 10 nm. For visible light, the human eye can recognize such spectral information as a color (Fig. 2 B,C). This phenomenon is selective to light polarization and direction and only occurs for transmitted TM-polarized or non-polarized light. TE-polarized and reflected illumination modes cannot provide color information (Fig. 2D). Unlike color filtering via plasmonic nanograting structures (with subwavelength hole or slit apertures)^12^,^13^,^14^,^21^, the wavelength at the resonance peak closely matches the structural periodicity of our nanostructures (Fig. 2A). In other words, the passage of light through this nanograting structure causes its geometric information to be converted into an electromagnetic spectrum. Interestingly, some color filters that use plasmonic subwavelength gratings can provide color information via their interfacial interactions rather than the air/metal surface because the corresponding spectral peaks are close to 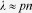
(SiO_2_, silica, and quartz substrates, refractive index *n *= 1.55 – 1.65 for the wavelength range from 200 to 500 nm). However, the transmission resonances that originated from the interface between the PMMA and the metal layer were significantly suppressed in our metal thin films when 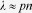
(*n *≈ 1.48, where *n* is the refractive index of the PMMA).

Numerous variations of the construction scheme can be adapted to obtain similar structures and achieve strong coupling efficiencies and sharp resonances. The SEM cross-sectional image presented in Fig. 3A depicts one such variation in which the silver nanowires are replaced with SiO_2_ while the rest of the structure remains the same. As seen from the measured transmission spectra presented in Fig. 3C, similar EOT effects and Fano resonances were observed. When the transparent SiO_2_ dielectric is used for the underlying nanowire array, the transmission efficiency at the resonance peaks is higher than for silver. These results suggest that the coupling efficiency and the EOT effect originate from the top nanostructured metal thin film. Therefore, the efficient subwavelength confinement of spatial modes in metal–insulator–metal (MIM) structures^22^ is not likely attributed to the high transmission efficiency in our structures. However, this material variation clearly affects the line shapes of the resonances. The Fano resonance line shape can be defined as 
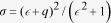
, where *q* is the phenomenological shape parameter and 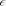 is the resonance energy distribution^23^,^24^. As the grating periodicity increases, the asymmetry of the Fano profiles (Fig. 3C) completely reverses (*q* > 0) relative to that of the silver nanowire structures (*q* < 0) (Fig. 2A). This trend may suggest that two coupling pathways exist, one in which the external field predominantly excites a continuous state that then couples to the discrete state and another in which the external field directly couples to the discrete state^25^,^26^. Our experimental results may suggest a new route for controlling the Fano resonance asymmetry and line shapes in plasmonic nanostructures.

The aforementioned method is not a unique means of forming curved edges in our thin-film nanostructures. Indeed, several techniques for thin-film deposition, such as chemical vapor deposition (CVD) and atomic layer deposition (ALD), provide uniformity and good step coverage by virtue of surface diffusion at elevated growth temperature. These techniques can be utilized for the deposition of the intermediate dielectric layers. Fig. 3 B,D represent nanostructures that were prepared by coating a 10 nm ZrO_2_ thin-film layer onto gold nanowire arrays at 170 °C via ALD, followed by depositing a 40 nm gold layer on top of the ZrO_2_-coated nanowire arrays. The cross-sectional profiles of these nanostructures exhibited an increased curvature (Fig. 3B). The transmission spectra revealed resonances corresponding to the periodicities of the structures, similar to previous results. However, the trend in transmission efficiency with increasing periodicity was reversed. This high curvature profile of the nanostructures strongly enhanced the performance of the large-period gratings. This finding indicates that these nanostructures may be capable of achieving high efficiencies even beyond the visible and near-infrared ranges. Hence, the coupling efficiency in our nanostructures strongly depends on the structural configuration. This structure offers a flexible platform whose composition, shape and size can be manipulated for further improvement of the coupling efficiency.

Because our nanostructured thin films behave so similarly to nanohole arrays, an understanding of the role played by surface plasmons can likely be used to understand and utilize these phenomena, as well. In particular, we can use such an understanding to expand the applications of surface plasmon optics. In addition to being spectral filters, nanohole arrays that are sensitive to refractive indices have been studied for use as sensing devices. To demonstrate the utility of our nanostructures for sensing applications, we verified the behavior of the transmission spectra as a function of the refractive index of the medium interfacing with the metallic thin-film surface. This refractive-index sensitivity is the basis of plasmonic detection. The spectral shifts were determined using NaCl solutions of various concentrations (5%, 10%, 15% and 20%) in deionized water (Fig. 4A). The measurements were performed under TM-polarized light at normal incidence. Several alcohols were also tested to further confirm the sensitivity of the plasmonic nanostructure (Fig. 4B). A general figure of merit, *FOM = S (nm RIU*^*-1*^*)/Γ (nm),* was used to quantify the performance of the plasmonic nanostructure in units of wavelength, where *S* is the sensitivity to refractive index and *Γ* is the resonance linewidth or FWHM^27^. A sharp dispersion was observed in our nanostructure system. The Fano resonance line widths were typically below 10 nm. Hence, these nanostructures can achieve a high resonance quality factor of approximately 100 at a grating periodicity of 600 nm. For sensing applications the Fano line shape can be affected by the refractive index changes. However the linewidth is a direct indicator of the quality of the resonances, which is more critically related to the coupling efficiency of the discrete and continuous states for the Fano resonances. Our measured figures of merit ranged from 80 to 130. This high sensitivity indicates that these structures are promising for use as plasmonic sensing devices that do not require prisms and laser sources. The grating periodicity can be tuned within the visible light range to detect specific chemical or biological substances. Although spectral instruments are required to distinguish modest spectral shifts, large shifts caused by changes in the refractive index or high molecular concentrations can be discerned by the human eye.

Our results clearly demonstrate that light can propagate with high efficiency through a metal thin film with a properly constructed structure. Depending on the nanostructural arrangement, the resonance frequency can be manipulated by varying the periodicity of the structure. For the visible light range, such nanostructures can be integrated with light display or capture systems for color display or spectral detection, respectively. In addition, the high refractive-index sensitivity of these nanostructures makes them suitable for use in chemical and biological sensing. The simplicity and flexibility of these structures and their fabrication methods promise new routes toward the development of nanostructured light-emitting diodes and photodiodes for lab-on-a-chip sensing devices.

## Fabrication Methods

Nanowire arrays are fabricated on Corning 1737 glass substrates using electron-beam lithography. In order to prevent electron charging distortion during the exposure, 7 nm aluminum thin film is thermally evaporated on spin-coated PMMA 950 resist. After the electron beam exposure and the developing of the resist, a 5 nm titanium layer was deposited by electron beam evaporation, followed by a 40 nm thick silver layer. Then lift-off process is used for the formation of nanowire array patterns on the glass substrate. A thin dielectric layer (1% PMMA 950 in MIBK) is spin-coated onto the nanowire-array-patterned substrate and then thermally baked at 180 °C of the temperature for 15 minutes to round the corners and edges and to smooth the surfaces. In the final step, a 40 nm sliver thin film layer is deposited by evaporation with a 2 nm titanium adhesion layer. We found that this very thin layer of titanium did not noticeably affect the spectral responses. The coating and baking of the polymer produces a smooth and curved layer around the nanowires that determine the final topography of the top metal thin film.

Thin film deposition techniques provide an alternative method to shape such structures. Here, we use atomic layer deposition (ALD) owing to its flexibility for a variety of dielectric materials. This thin film deposition technique produces thin and conformal films with accurate control of the thickness to cover the entire surface of the substrates. During the growth, surface diffusion also can smooth surface, and round edges and corners. There is no special consideration to use ZrO_2_ in our experiments. Other dielectrics such as Al_2_O_3_, HfO_2_, SiO_2_ and so on are also good candidates for coating the nanowire array structures. Difference in their structural (crystallinity) and physical properties (refractive index) may affect the optical responses that would be worthy of further investigation. In the ALD process, tetrakis (dimethylamino) zirconium [(CH_3_)_2_N]_4_Zr (TDMAZr) and the oxidizer of deionized water are used as precursors for ZrO_2_ deposition. Ultra high purity nitrogen is used as the carrier and purge gas. The chamber pressure for the ALD deposition is 6–7 mTorr. The ZrO_2_ pulse time is 150 ms and purge time is 20 seconds for both TDMAZr and water, resulting in an approximate deposition rate of 0.1 nm per cycle. The deposited thickness of ZrO_2_ is 10 nm for 100 cycles.

## Additional Information

**How to cite this article**: Xiao, B. *et al*. Enhanced optical transmission and Fano resonance through a nanostructured metal thin film. *Sci. Rep.* **5**, 10393; doi: 10.1038/srep10393 (2015).

## Figures and Tables

**Figure 1:**
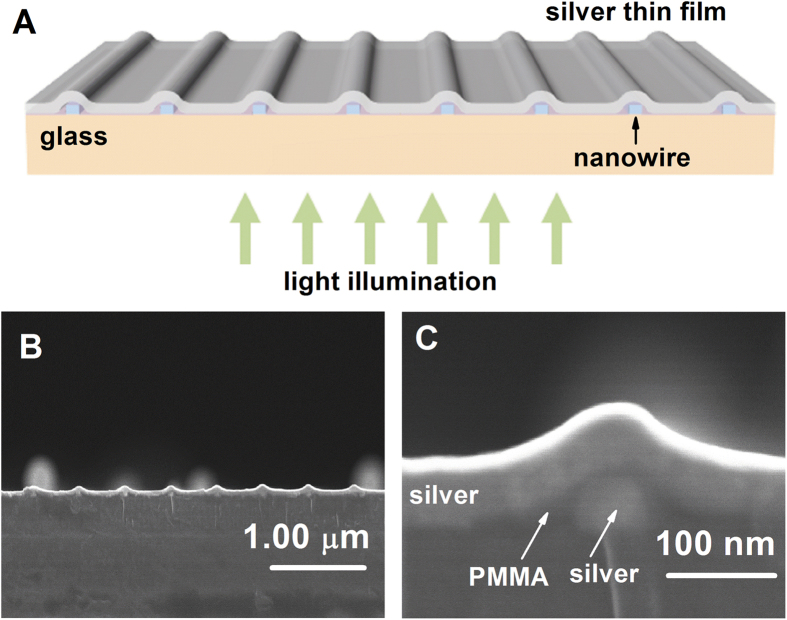
(**A**) Schematic diagram of the metal thin-film nanograting. (**B** and **C**) SEM images of a cross section of the structure. The nanowire array has a period of 600 nm, a height of 40 nm and a nanowire width of 60 nm. The PMMA thickness is approximately 10 nm after baking. The top metal layer is approximately 50 nm thick.

**Figure 2:**
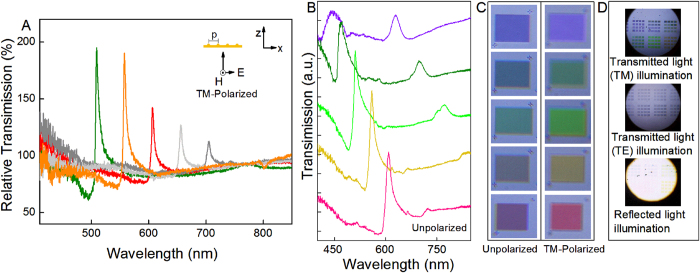
(**A**) Experimental optical transmission spectra for the metal thin-film nanograting under TM illumination for structures with various periods of 500, 550, 600, 650 and 700 nm; these periods correspond to the resonance peaks observed in the figure from left to right. (**B**) Experimental transmission spectra obtained under non-polarized light. (**C**) The corresponding color images acquired using a microscope under transmitted non-polarized and TM-polarized light. From top to bottom, the images and curves presented in (**B**) and (**C**) correspond to grating periods of 400, 450, 500, 550 and 600 nm. (**D**) Color microscopy images of a variety of nanostructure arrays under various modes of illumination: transmitted TM-polarized light, transmitted TE-polarized light and reflected light.

**Figure 3:**
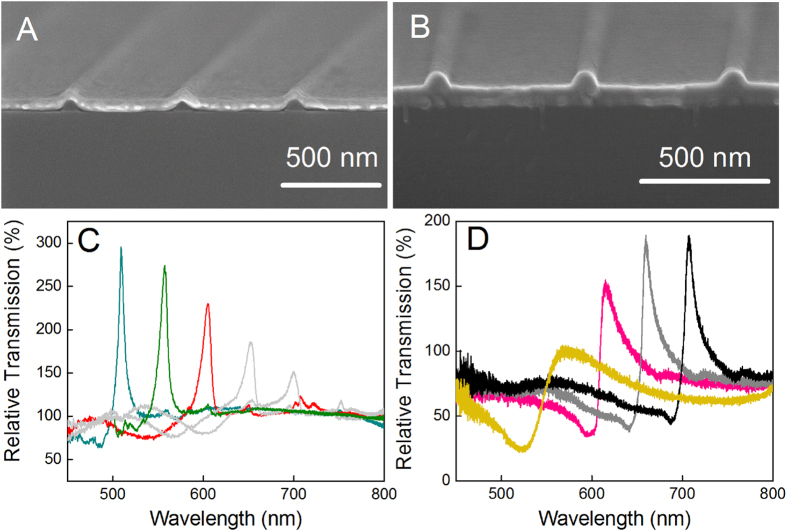
(**A**) and (**B**) SEM cross-sectional images and (**C**) and (**D**) experimental optical transmission spectra of metal thin-film nanogratings under TM-polarized illumination for various grating periods: 500, 550, 600, 650 and 700 nm and 550, 600, 650 and 700 nm, respectively, corresponding to the resonance peaks observed in the plots from left to right. Fig. 3 A,C represent structures with SiO_2_ nanowire arrays. Fig. 3 B,D represent structures with a ZrO_2_ dielectric layer prepared using the ALD method.

**Figure 4:**
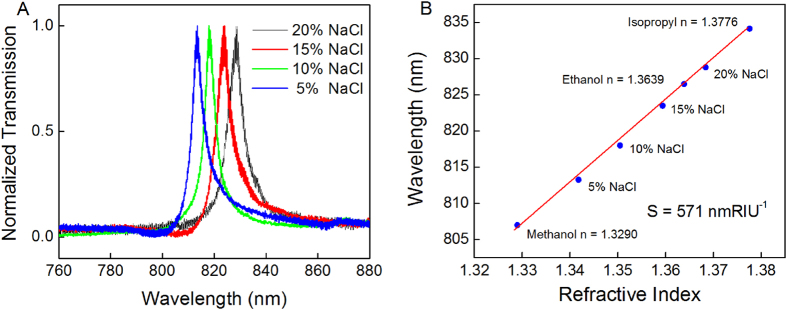
(**A**) Transmission spectra for a nanostructured silver thin film obtained with the film in contact with NaCl solutions of various concentrations. The periodicity of the grating (*p*) was 600 nm. The concentrations of the NaCl solutions were 5%, 10%, 15% and 20% in deionized water, corresponding to refractive indices of 1.3418, 1.3505, 1.3594, and 1.3684, respectively. (**B**) The dependence of the wavelength shift on the refractive index (*p* = 600 nm). The sensitivity of the nanostructured thin film was determined via linear fitting. The results for ethanol (95%, n = 1.3639), methanol (n = 1.3290) and isopropyl alcohol (n = 1.3776) are also indicated on the curve^28^.
